# Aquaporin 5 Polymorphisms and Rate of Lung Function Decline in Chronic Obstructive Pulmonary Disease

**DOI:** 10.1371/journal.pone.0014226

**Published:** 2010-12-03

**Authors:** Nadia N. Hansel, Venkataramana Sidhaye, Nicholas M. Rafaels, Li Gao, Peisong Gao, Renaldo Williams, John E. Connett, Terri H. Beaty, Rasika A. Mathias, Robert A. Wise, Landon S. King, Kathleen C. Barnes

**Affiliations:** 1 Department of Medicine, School of Medicine, Johns Hopkins University, Baltimore, Maryland, United States of America; 2 Bloomberg School of Public Health, Johns Hopkins University, Baltimore, Maryland, United States of America; 3 Division of Biostatistics, School of Public Health, University of Minnesota, Minneapolis, Minnesota, United States of America; Ludwig-Maximilians-Universität München, Germany

## Abstract

**Rationale:**

Aquaporin-5 (AQP5) can cause mucus overproduction and lower lung function. Genetic variants in the *AQP5* gene might be associated with rate of lung function decline in chronic obstructive pulmonary disease (COPD).

**Methods:**

Five single nucleotide polymorphisms (SNPs) in *AQP5* were genotyped in 429 European American individuals with COPD randomly selected from the NHLBI Lung Health Study. Mean annual decline in FEV_1_ % predicted, assessed over five years, was calculated as a linear regression slope, adjusting for potential covariates and stratified by smoking status. Constructs containing the wildtype allele and risk allele of the coding SNP N228K were generated using site-directed mutagenesis, and transfected into HBE-16 (human bronchial epithelial cell line). AQP5 abundance and localization were assessed by immunoblots and confocal immunofluoresence under control, shear stress and cigarette smoke extract (CSE 10%) exposed conditions to test for differential expression or localization.

**Results:**

Among continuous smokers, three of the five SNPs tested showed significant associations (0.02>*P*>0.004) with rate of lung function decline; no associations were observed among the group of intermittent or former smokers. Haplotype tests revealed multiple association signals (0.012>*P*>0.0008) consistent with the single-SNP results. In HBE16 cells, shear stress and CSE led to a decrease in AQP5 abundance in the wild-type, but not in the N228K AQP5 plasmid.

**Conclusions:**

Polymorphisms in *AQP5* were associated with rate of lung function decline in continuous smokers with COPD. A missense mutation modulates AQP-5 expression in response to cigarette smoke extract and shear stress. These results suggest that *AQP5* may be an important candidate gene for COPD.

## Introduction

COPD is the fourth leading cause of death in the United States and the fifth leading cause of death worldwide and its prevalence is expected to increase in coming decades.[1], [2] The overwhelming majority of COPD is caused by environmental exposures. In the United States, this exposure is primarily cigarette smoke; however only 15% of smokers develop COPD,[3] suggesting an important role for genetic susceptibility.

COPD is characterized by abnormal mucous production which may promote bacterial adhesion and may impair bacterial clearance leading to chronic inflammation and irreversible airflow limitation.[4], [5] Aquaporins are water-specific membrane channel proteins and aquaporin 5 (AQP5) is found in airway epithelial cells, type I alveolar epithelial cells and submucosal gland acinar cells in the lungs where it plays a key role in water transport.[6] Decreased expression of human AQP5 has been associated with mucus overproduction in the airways of subjects with COPD and lower lung function.[7] Furthermore, smoking has been shown to attenuate the expression of AQP5 in submucosal glands of subjects with COPD.[7] These data support a potential role of AQP5 in severity of airflow obstruction in COPD and suggest that the expression of AQP5 may be modified by smoking exposure.

*AQP5* is a single copy gene on human chromosome 12q13.[8] A single nucleotide polymorphism (SNP) in intron 3 (rs3736309) has been associated with the presence of COPD in a Chinese population, but not with cross-sectional measures of lung function or COPD severity.[9] Whether polymorphisms in AQP5 correlate with the decline of pulmonary function, a trait associated with the development and progression of COPD, is unknown. In this study, we examined associations between genetic variants in the *AQP5* gene and rate of lung function decline in a randomly selected subset of the multicenter NHLBI-supported Lung Health Study (LHS) cohort. Identifying pathways and novel molecular targets that modify the clinical course of disease is fundamental to developing preventive strategies and novel therapies.

## Methods

### Ethics Statement

This study has been approved by the Johns Hopkins University Institutional Review Board. Written informed consent for research was obtained from all participants of the LHS and consent for this analysis was waived because the research involved no additional risk to subjects, and the data used was de-identified. Findings from this manuscript were previously presented in abstract form.

### Study Subjects

We randomly selected 429 European Americans of the LHS for whom DNA was available. The LHS was a multicenter (10 centers) randomized clinical trial aimed to determine whether a program of smoking intervention and use of an inhaled bronchodilator could slow the rate of decline in pulmonary function over a 5-year follow-up period. Details of LHS methods have been described extensively.[10]–[12] LHS inclusion and exclusion criteria included the following: Patients were all active smokers between the ages of 35 and 60 with mild to moderate airflow obstruction defined as an FEV_1_/FVC ratio less than 0.7, and an FEV_1_ between 50%–90% predicted. Lung function was measured annually over five years.[11], [13], [14] Lung function data from Annual Visit 1 to Annual Visit 5 was used for the current analyses and has been shown to have a good linear fit in previous LHS analyses.[11], [14] Subjects with <3 annual lung function measurements were excluded from analysis (n = 15), resulting in a final group of 414 subjects with data available.

### SNP Selection and Genotyping

**Figure 1:**
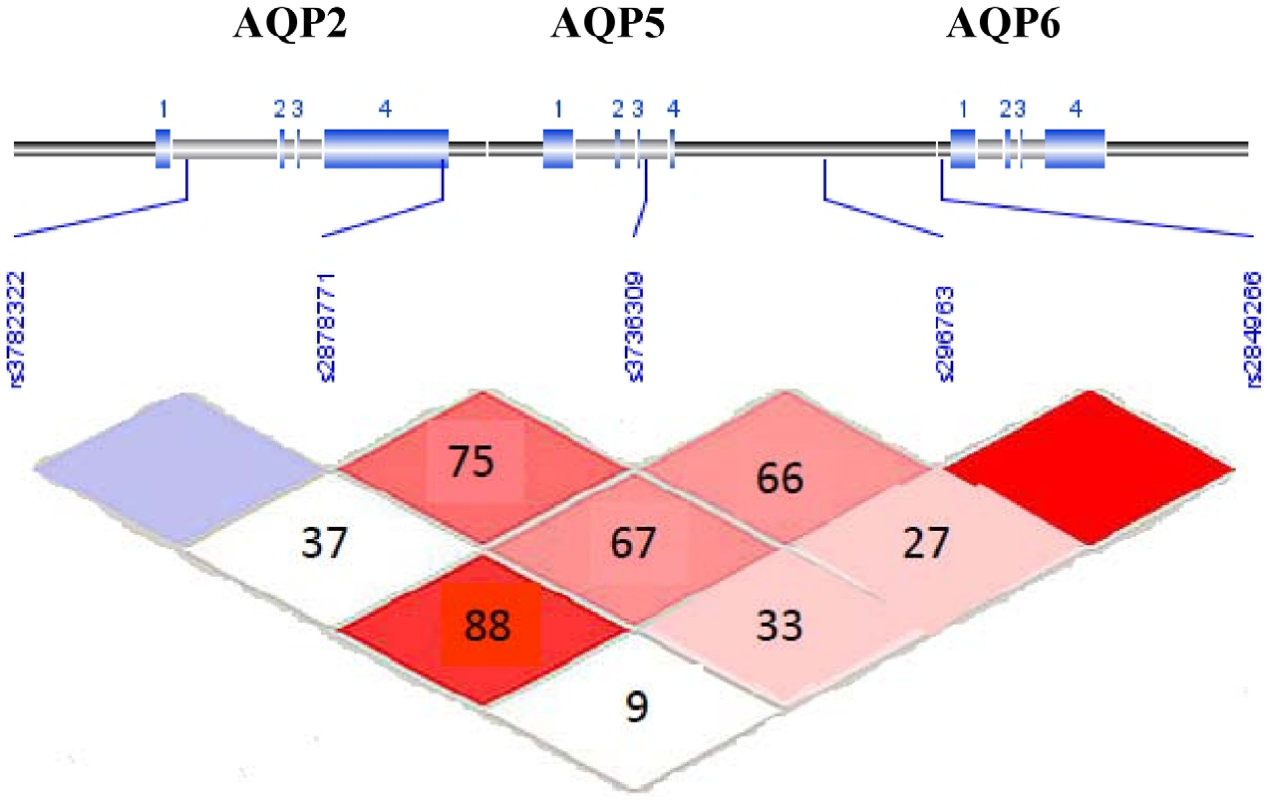
Linkage Disequilibrium for 5 SNPs in the *AQP5* Gene in European American participants with COPD. Pairwise LD in subjects is represented as red squares for strong LD, blue squares for non-significant LD, and white squares for little or no LD. LD blocks are identified as noted.

Single nucleotide polymorphisms (SNPs) in the *AQP5* gene were selected from Goldenpath (http://genome.ucsc.edu/) and/or NCBI (http://www.ncbi.nlm.nih.gov/). As AQP5 is within the gene cluster of 3 AQP genes (including AQP2 and AQP6; see **Figure 1**), to test whether AQP5 gene itself versus loci in surrounding regions influence disease risk, a total of five SNPs spanning 21,000 bp on human chromosome 12q13 with an average inter-SNP distance of 5.25 kb (ranging from 3.3–7.1 kb) were selected for genotyping using the rationale described below, and genotyped on the Illumina™GoldenGate platform. The study was designed in 2005, prior to conventional LD-tagging approaches for SNP selection. Consequently using standard methodology available at that time, criteria used for SNP selection included 1) SNPs approximating an inter-SNP distance as close to 5 kb as were available at the time of the dbSNP Build 124; 2) representation of SNPs in the promoter, coding and 3′UTR regions; and 3) SNPs with acceptable design scores according to the Illumina Assay Design Tool for genotyping on the Illumina™ GoldenGate platform. Priority for selecting SNPs included: 1) regulatory and coding SNPs; 2) highly polymorphic SNPs, preferably ≥10% minor allele frequency; 3) validated SNPs; and 4) SNPs at intron/exon boundaries.

### Statistical Analysis

Mean annual decline in post-bronchodilator FEV_1_ % predicted (%predFEV_1_)(11) was calculated as a linear regression slope using all available time points. Linear regression models were used to adjust for potential confounders, including 1) baseline characteristics: smoking history (pack-years), age, sex, %predFEV_1_ and airway reactivity (AR); 2) change in body mass index (BMI); and 3) smoking status at Year 5 of the observation period, defined as 1) ‘continuous smoker’ or 2) ‘intermittent or former smokers’. Forward and backward selection was used to develop a parsimonious model. Age, baseline lung function, AR, and change in BMI were included in the final regression analyses. Interaction term for smoking and genetic effect were tested using PLINK. There was significant interaction (*P*<0.05) for at least one of the five SNPs, so analyses were stratified by smoking status. Residuals from this regression were used in an additive genetic model for each SNP where the most common homozygote for each SNP served as the reference category. Each SNP locus was evaluated for Hardy-Weinberg equilibrium. Pairwise linkage disequilibrium (LD) based on the D' statistic was measured using Haploview.[15] LD blocks were defined using their default algorithm.[16] Sliding windows of 2–4 adjacent SNPs were used to test for association. All analyses were performed with StataSE, version 8.0 (Stata Corp, College Station, TX) and PLINK.[17]

### Functional Analysis

#### SNP information

A non-synonymous, but non-validated SNP (rs41495048) was subsequently identified from dbSNP build 128 (www.ncbi.nlm.nih.gov). It did not meet acceptable design criteria for genotyping on the Illumina™ GoldenGate platform or the alternative TaqMan™ probe-based, 5′ nuclease assay with minor groove binder (MGB) chemistry (ABI, Foster City, CA). However, given that this SNP is flanked by SNPs 3 and 4 (**Table 1** **and** **Figure 1**), which were significantly associated with lung function decline, and the only non-synonymous SNP identified at the time of this analysis, it was selected for further functional studies.

**Table 1:** Location, minor allele frequency, and type of selected *AQP5* SNPs.

SNPID	SNP	Position	Region	European American	
				Minor Allele	MAF
1	rs3782322	48631567	promoter	A	0.09
2	rs2878771	48638660	promoter	C	0.19
3	rs3736309	48644321	intron (boundary)	G	0.17
4	rs296763	48649281	downstream	G	0.24
5	rs2849266	48652567	downstream	C	0.08

#### Plasmid Generation

Human AQP5 was inserted into the *EcoR1* site as previously described.[18]. In order to create N228K AQP5, degenerate primers were created and N228K human AQP5 was amplified by PCR using QuikChange Site-Directed Mutagenesis Kit (Stratagene) according to manufacturer's recommendations. Therefore both the wild-type and N228K plasmid have the same backbone and both express the hemagglutinin tag.

#### Cell culture and transfection

Immortalized human epithelial cells, which are endogenously AQP5 null (16HBE cells, gift of Gary Curtting) were grown in chamber slides to 50–60% confluence, and transiently transfected (1 µg/well) with either wild – or SNP-expressing HA-tagged human AQP5 cDNA using FuGENE 6 (Roche) (1.5 µl) according to the manufacturer's recommendations.

#### Cell exposures

Shear stress was applied to 16HBE cells transfected with either wildtype hAQP5 or N228K hAQP5 plasmid as described previously.[19] Briefly, low levels of flow was used to create shear forces of 0.5–1 dyne/cm^2^, to estimate the shear produced by airflow through the tracheobronchial tree. CSE was prepared as previously published.[20], [21] Smoke from 1 research cigarette, 1R5F (University of Kentucky) was bubbled through 10 ml of PBS at 3000 rpm. The CSE was filtered through a 0.2 µm sterile filter (Corning, NY) and pH adjusted to 7.0. The CSE was diluted to 10% of initial concentration and used within 20 min to avoid breakdown of substances.

#### Immunoblotting and immunofluorescence

Total protein concentrations of the samples were determined by the BCA assay with bovine serum albumin as the standard. Depending on the experiment, 10 to 50 µg of total protein in 1.5%(w/v) SDS was loaded per lane and separated on 12% SDS-PAGE gels prior to transfer to a nitrocellulose membrane. Immunoblotting and blot visualization were performed as described.[18] Affinity purified antibody against the carboxy-terminus of human AQP5 were generated in our lab as previously described.[22] This antibody was used to detect both the wild-type as well as the N228K protein, as the single nucleotide switch did not affect the affinity of the antibody to the protein. Equivalence of samples was confirmed by probing for actin (Upstate Biotechnology). The bands are shown from a total of three different repeated experiments, and the densitometry was collected by comparing the changes in all of the samples to their controls, therefore representing the sum total of 3 different experiments, with replicates within each experiment. Confocal laser microscopy (SP5, Leica) was performed on cells grown in uncoated chamber slides (Falcon) using antibodies against human AQP5 and goat anti-rabbit secondary antibodies (Alexa 488; Molecular Probes). Merged images for analysis of double labeling or z-planes were reconstructed by differential interference contrast images.

## Results

Clinical characteristics of subjects have been published previously[23] and are presented in **Table 2** **.** There were no statistical differences in baseline characteristics between those included in the final analyses (n = 414) compared to those excluded because they had >2 missing data points in lung function (n = 15, data not shown). Distributions of baseline characteristics and lung function measurements were also similar to those in the full LHS cohort from which the subset was randomly selected (n = 5,887, data not shown).

**Table 2:** Patient characteristics.

	European Americans(n = 414)
***Baseline Characteristics***	
Mean age (SD)	48.6 (7.0)
Male, %	65.0
BMI, kg/m^2^	25.4 (3.6)
Smoking (pack-years)	40.5 (19.2)
Pre-BD FEV_1_ % predicted, %	76.3 (6.4)
Post-BD FEV_1_ % predicted, %	79.2 (6.2)
***Longitudinal Characteristics*** ***at 5 years***	
Smoking history, %	
Continuous	50
Intermittent	31
Sustained quitter	19
Post-BD ΔFEV_1_, %/yr	−0.74 (1.63)

BD – bronchodilator.

BMI – body mass index.

SD – standard deviation.

### Single-marker analyses

All SNPs were in Hardy-Weinberg Equilibrium (HWE). All results of the two-point tests for association between *AQP5* markers and %predFEV_1_ decline are presented in **Table 3**, highlighting significant associations (*P*<0.05) between rate of lung function decline and three of the five SNPs tested in continuous smokers. There was no association between tested SNPs and lung function decline in the group of intermittent or former smokers. Results were similar when intermittent and former smokers were analyzed separately (data not shown).

**Table 3:** Association of *AQP5* polymorphisms with lung function decline in continuous smokers and intermittent or former smokers.

			Continuous smokers		Intermittent or former smokers	
SNPID	SNP	Minor Allele	Beta	p-val	Beta	P-val
1	rs3782322	A	−0.072	0.782	0.075	0.807
2	rs2878771	C	0.547	**0.004**	−0.074	0.716
3	rs3736309	G	0.502	**0.024**	0.119	0.544
4	rs296763	G	−0.430	**0.012**	−0.077	0.679
5	rs2849266	C	0.161	0.542	0.293	0.340

### Haplotype Analyses

**Figure 2:**
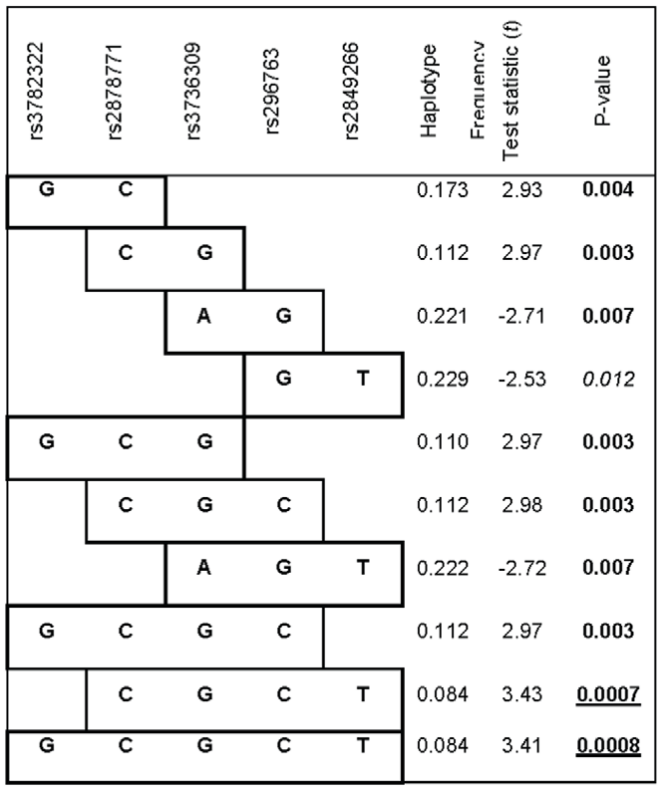
Haplotype Analysis. Using the sliding window approach of 2–4 SNPs/window beginning with the first (5′) marker, and working across the gene, one marker at a time, the results of significant (defined as *P*<0.05) haplotype signals in continuous smokers are shown.

Strong linkage disequilibrium was observed for only one out of four pairings of contiguous SNPs and no LD blocks were identified in this gene under the Gabriel algorithm[16] **(** **Figure 1** **),** therefore a systematic sliding window approach was implemented, considering haplotypes of 2–4 SNPs beginning with the first (5′) marker, and working across the gene. Haplotype tests further support the single SNP results with stronger P-values that appear to highlight the background haplotype likely carrying the potentially untyped but tagged causal SNP; multiple windows of signal that include the single SNPs with P<0.05 described above **(** **Figure 2** **).** The haplotype CGCT (rs2878771, rs3736309, rs296763, rs2849266) showed the strongest association with rate of lung function decline of all haplotypes tested (*P* = 0.0007), and provides a stronger signal than any of the single SNPs, albeit with the same direction of effect (i.e., protective) on lung function decline.

### Functional Analysis

**Figure 3:**
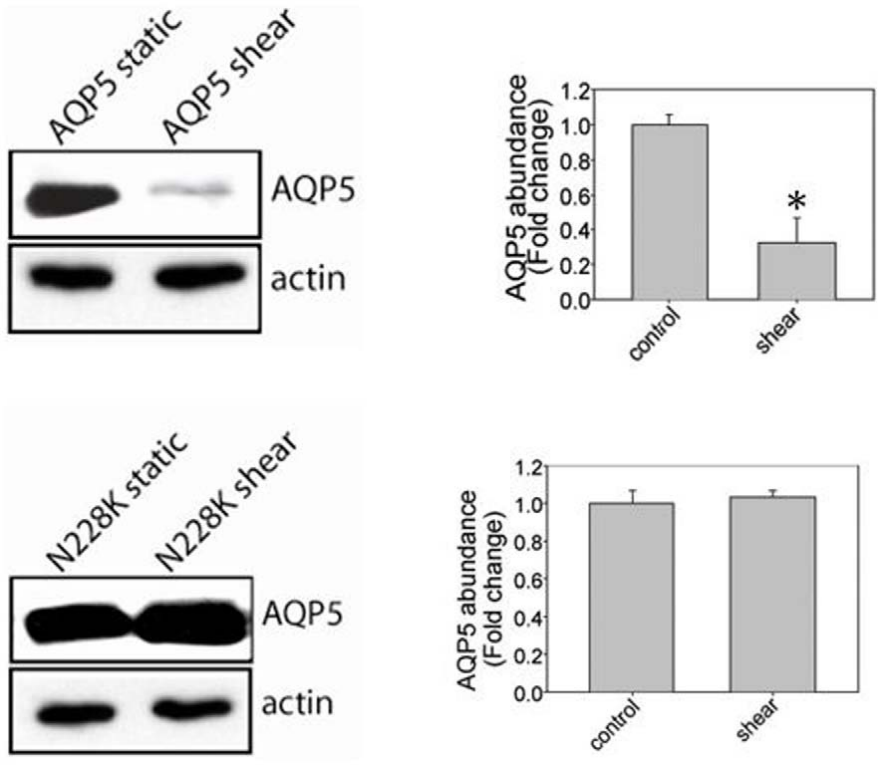
Exposure to shear stress leads to a decrease in wild-type AQP5 but no change in N228K AQP5 (n = 3; * p<0.05 with ANOVA one-way).

**Figure 4:**
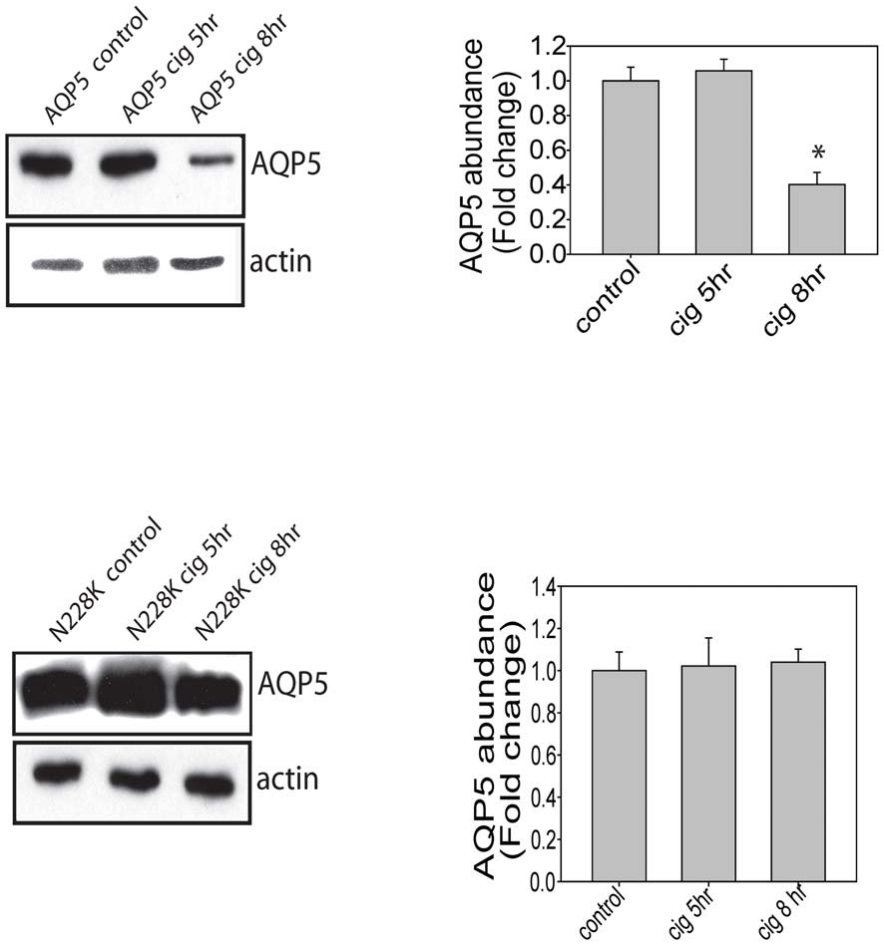
Eight hours of 10% CSE leads to a decrease in wild-type AQP5 but no change in N228K AQP5 (n = 3; * p<0.05 with ANOVA one-way).

**Figure 5:**
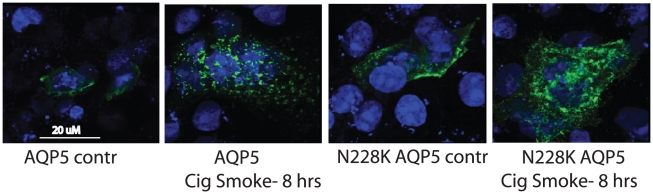
Both wild-type and N228K AQP5 are present on the membrane under control conditions. CSE leads to internalization of both.

Under control conditions, there was similar abundance in both the wild-type AQP5 plasmid and N228K AQP5 plasmid. Shear stress led to a decrease in AQP5 abundance after two hours; however, similar changes were not observed in N228K AQP5, which showed no change in abundance after shear stress **(** **Figure 3** **)**. To test if the N228K AQP5 abundance was different from wildtype AQP5 in response to 10% CSE, 16HBE cells transfected with wildtype hAQP5 or N228K hAQP5 were exposed to either PBS or 10% CSE. CSE exposure led to decreased wild-type hAQP5 abundance at eight hours, and again there was no change in the N228K hAQP5 plasmid abundance **(** **Figure 4** **)**. Localization in response to these stimuli was not altered in response to either shear (data not shown) or CSE **(** **Figure 5** **)**, indicating that in 16HBE cells, the N228K AQP5 altered AQP5 abundance in response to physiologic stimuli (such as shear stress) as well as pathologic stimuli (such as cigarette smoke extract) without changes in distribution.

## Discussion

We identified three SNPs in the *AQP5* gene significantly associated with rate of lung function decline in a European American population of continuous smokers with COPD, which was further supported by haplotype analysis. Furthermore, a single non-synonymous SNP, flanked by SNPs which were significantly associated with rate of lung function decline, has been identified in *AQP5*; with this information, we created a human-AQP5 plasmid using site-directed mutagenesis. Wildtype hAQP5 and N228K hAQP5 responded differently to both the physiologic stimulus of low level shear stress, as well as pathologic exposure of cigarette smoke exposure (CSE) in human bronchial epithelial cells. Collectively our results implicate *AQP5* as a novel candidate gene for rate of lung function decline and COPD.

Our strongest single-marker association was for a marker in the promoter region, wherein the C allele at rs2878771 was associated with a 0.55% per year attenuation (i.e., protection) in loss of FEV_1_ % predicted in continuous smokers, a considerable effect size. In other words, over a 40 year period, a person homozygous for the C variant will be predicted to experience a decline in FEV_1_ % predicted of 44% *less* compared to a homozygote for the major allele. Thus over time, this genetic variant could substantially impact disease progression. For example, given a MAF of 0.19, as seen in our cohort, we might extrapolate 4% of the population to be homozygote for the minor allele (CC), 31% to be heterozygote and 66% to be homozygote for the major allele. Even after adjusting for smoking status, this genetic variant could explain the difference between mild and severe COPD under current GOLD criteria.[24]

We identified two additional SNPs in the *AQP5* gene significantly associated with rate of lung function decline, including a SNP in intron 3 (rs3736309), where carriers of the G allele had a lower prevalence of COPD in a Chinese population.[9] Our results confirm that the G allele may confer some protection from COPD as the G allele in rs3736309 was associated with a significant attenuation in lung function decline among smokers.

Though our strongest haplotype signals span the gene (∼3 kb) rendering it difficult to narrow the association to a causal variant, to facilitate identifying variants/mutations in *AQP5* that may alter expression of AQP5, the non-synonymous SNP in exon 4 of *AQP5*, which results in an amino acid change (Asn to Lys) was created in a human-AQP5 plasmid using site-directed mutagenesis. This SNP was selected because it was the only non-synonymous SNP identified in *AQP5* at the time of the study and was flanked by two intronic SNPs, including rs3736309, used in our analysis and found to be associated with rate of lung function decline. Our results show that this functional mutation alters hAQP5 abundance in HBE-16 cells treated with CSE and low level shear stress. The wildtype hAQP5 abundance decreases; however, no similar reduction was observed in the N228K hAQP5 treated with low level shear stress and CSE at two and eight hours, respectively. This differential regulation of protein expression and protein stability to these stimuli could contribute to functional differences in lung function seen in individuals with COPD.

COPD is influenced by genetic and environmental factors and the interaction between genetic and environmental influences are likely to be important in the pathogenesis of COPD.[25] Cigarette smoking is the major environmental determinant of COPD and genetic and smoking interactions have been associated with lung function in COPD and other chronic lung diseases.[26]–[29] We found effects of genetic variants in *AQP5* are stronger in continuous smokers than among those who have quit smoking or smoke intermittently. A specific mechanism by which this interaction could occur is suggested by *in vitro* studies showing smoking attenuates expression of AQP5 in submucosal glands of subjects with COPD.[7] This decreased expression of human AQP5 has been associated with mucus overproduction in the airways of subjects with COPD and lower lung function.[7] Our *in vitro* studies confirm wildtype hAQP5 abundance decreases after exposure to CSE and low level shear stress.

In addition to mucous hyper-secretion, another potential mechanism by which *AQP5* may lead to lung function loss in subjects with COPD includes a predisposition to airway reactivity as *aqp5* −/− mice have been shown to be hyperresponsive to bronchoconstriction by cholinergic stimulation.[30] Increased airway reactivity characterizes both asthma and COPD and has been previously associated with longitudinal changes in lung function in the LHS cohort.[14]

In recent years, there has been interest in the ‘common variant/multiple disease’ hypothesis and suggests certain disease genes may contribute to related clinical phenotypes.[31] For example, previous literature suggests that there are overlapping associations between several genes and chronic lung diseases, including asthma, cystic fibrosis and COPD.[32]–[36] In addition, AQP5 has been suggested to be implicated in asthma pathogenesis,[30], [37], [38] however, whether AQP5 polymorphisms contribute to rate of lung function decline in other lung diseases, such as asthma, remains to be determined.

In summary, our results demonstrate *AQP5* polymorphisms are associated with rate of lung function decline among continuous smokers with COPD in the LHS. A missense mutation may be involved in regulation of AQP-5 expression in response to cigarette smoke extract and shear stress, however the functional variant in human *AQP5* responsible for differential rate of lung function decline and its mechanism of action is yet to be identified. Moreover, the relative lack of information with regard to non-synonymous SNPs in the *AQP5* gene points to the importance of deep re-sequencing. Collectively, these results identify *AQP5* as a novel target in COPD and lung function loss.
